# Mobile asthma apps for emerging adults transitioning to college: A scoping review

**DOI:** 10.1016/j.hctj.2025.100112

**Published:** 2025-07-17

**Authors:** V. Ann Andreoni, Margaret C. Delaney, Susan McNamee, Elizabeth Huggins, Barbara Velsor-Friedrich

**Affiliations:** a Marcella Niehoff School of Nursing, Loyola University Chicago, Health Sciences Campus, 2160 S. First Avenue, Building 125, Maywood, IL 60153, United States; b Student Wellness Center,Loyola University Chicago, Health Sciences Campus, 2160 S. First Avenue, Building 125, Maywood, IL 60153, United States; c Health Sciences Library, Loyola University Chicago, Health Sciences Campus, 2160 S. First Avenue, Building 125, Maywood, IL 60153, United States

**Keywords:** Scoping review, Self-management, Asthma app, College students, Emerging adults

## Abstract

**Background:**

Limited data exists to direct evidence-based asthma self-care for emerging adults as they transition from pediatric to adult health care. This transition often occurs in the college setting, apart from usual parental and health care support systems. Given the chronic, episodic nature of asthma, it is imperative that emerging adults have essential tools to maintain disease control.

**Objective:**

A scoping review was conducted to discover if mobile applications (apps) for asthma self-care and management exist for college students between 17 and 25 years of age.

**Results:**

Research studies (n = 456) from peer-reviewed journals published between 2015 and 2023 were analyzed. As the search revealed no published articles that met the inclusion criteria for age and educational status, the criteria were revised to eliminate the requirement for college enrollment. Six published articles evaluating 3 separate asthma apps with similar age ranges were identified but without the same educational transition needs.

**Discussion:**

The studies reviewed suggested that app use had a positive effect on participants, including ease of information access and improved disease knowledge, symptom control, and/or symptom self-management. Limitations included small sample sizes, participant age variability, and lack of studies on needs specific to emerging adults. Small sample sizes, potential bias due to self-report variability, and inherent bias in cell phone availability in disadvantaged populations limit generalizability.

**Conclusion:**

Mobile asthma apps can facilitate transitional care in the emerging adult population. Although this scoping review suggests improved health outcomes with app use, there remains a need for future research on emerging adults with asthma transitioning to college.

## Highlights

• Uncontrolled asthma is a major contributor to the economic burden of health care.
• Available mobile asthma management applications for emerging adults are lacking.
• College transition may leave emerging adults unprepared for asthma self-management.
• Evidence suggests that use of an asthma app during the transition to college may contribute to better health outcomes.

## 1 Introduction

Asthma is a serious chronic disease that causes variably reversible airflow obstruction in the lower airways due to inflammation and smooth muscle tightness that imposes a significant economic and health burden on people of all ages.^1^, ^2^ In the US, asthma prevalence in those aged 15–19 years is 9.7 %, and 12.0 % in the 20-to-24-year age group.^1^ The prevalence and severity of the illness persists for many into adolescence, young adulthood, and beyond.^3^ Uncontrolled asthma affects approximately 50 % of children and 62 % of adults which leads to increased use of health care resources.^4^, ^5^ Asthma is such a substantial public health concern that major national and global health organizations spend significant time, energy and dollars on initiatives designed to reduce asthma attacks, emergency department (ED) visits and hospitalizations in order to improve the quality of life for those with the disease.^2^, ^5^, ^6^

Since the advent of the 21st century, experts have increasingly recognized that emerging adulthood, defined as the period between the ages of 18 and 29 years, is a separate developmental stage from adolescence with its own tasks and challenges.^7^ As adolescents with chronic diseases such as asthma enter this phase of life, an important task is to transition from family-centered pediatric health care to person-centered adult health care.^8^ Ideally, this happens before or at age 18, though for a variety of reasons, the transition may be delayed.^9^ However, a successful health care transition is essential to ensure that emerging adults are adequately prepared for independent self-care so they can avoid preventable complications and achieve the best quality of life.

The unpredictable nature of asthma episodes produced by exposure to a wide range of physical, emotional, and environmental triggers often affects all aspects of life.^10^ The episodic nature of asthma requires frequent planned and unplanned health care visits, self-monitoring for symptoms and knowledge on how to implement a personalized Asthma Action Plan. Emerging adults with asthma must frequently engage in advanced planning and active decision-making to manage their health effectively. This all comes at a time when they are coping with normal developmental changes in their lives such as getting their first jobs or starting college.

The transition period from parent-delivered care to self-management is a particularly vulnerable time for the emerging adult population.^3^ Of note, asthma-related deaths peak in older adolescents and young adults in comparison to those under 12 years of age. It is reported that the reasons for these deaths are largely associated with poor adherence to asthma management,^3^, ^11^ a risk that increases when transition of care does not occur smoothly.

Using a grounded theory approach, Velsor-Friedrich and Hogan interviewed college students with asthma about their transition from parent-managed to self-managed asthma care.^12^ The participants were startled to find themselves responsible for managing their condition once they lived apart from their families and usual health care providers, and were often unaware of the procedure for obtaining health care or getting prescriptions refilled. They conveyed their experience as an unexpected leap into an unfamiliar situation that they did not feel they were prepared for by their parents or care providers.^12^ Velsor-Friedrich and Hogan’s work generated the Theory of Being Unprepared which describes how college students in emerging adulthood often struggle to acquire asthma self-management skills. The theory suggests that college students with asthma have unique needs that may differ from those who are not attending college that may not be adequately addressed by currently available apps designed for younger or older age groups.^12^

In recent years, electronic health (eHealth) modalities have become part of the health care landscape. eHealth, defined as “a set of technologies applied with the help of the internet, in which health care services are provided to improve quality of life and facilitate health care delivery”,^13^ has been evaluated in patients with asthma.^10^ One method that has been studied is the use of mobile health phone and tablet applications (apps), some of which include functions for medication and symptom tracking, resource identification, health education, data collection, and information storage. The frequent use of mobile phones and tablets among emerging adults as well as their familiarity with these devices’ functions makes eHealth an attractive medium for obtaining and managing information in this age group.

The number and type of mobile apps that are focused on asthma have increased significantly since they first became available in the 2000s. Primary purposes of the apps range from serving as a patient-provider interface to patient-facing devices for tracking symptoms and medication doses as well as accessing education on asthma-related topics. Apps for patients with asthma have been tested in both pediatric and adult populations,^14^, ^15^, ^16^ but it is difficult to extrapolate the use of the technology to groups other than those examined, as studies lack consistency in not only participant demographics, study purposes, app design and functionality, but in desired outcomes.^10^ Some age groups, such as emerging adults, have received little attention from researchers, with their data incorporated into both pediatric and adult literature making it impossible to understand their unique needs, ideas and viewpoints.

Asthma self-management apps have been periodically evaluated for quality and substance. Huckvale et al.,^17^ initially reviewed the functionality and quality of asthma apps available in the English language. Of the 103 apps reviewed, 56 were educational in nature and 47 supplied tools for asthma management. None of the apps reviewed had both types of features, and, disturbingly, some were found to contain inaccurate information. A subsequent review by Huckvale et al.,^18^ found that although the number of available apps had doubled, the quality of the information remained poor, and app functionality was limited. Farzandipour et al.,^15^ reviewed 10 apps (7 for adults and 3 for adolescents) to assess the effectiveness of apps designed to promote asthma self-management. Nine of the ten studies in this series reported positive effects on the health of app users. Talavera (unpublished data) sought to locate apps that would specifically appeal to college students. Two adult apps that were available for iOS and Android devices were identified: Propeller Health and AsthmaMD. Both apps had desirable features (symptom tracking, Asthma Action Plan access) as well as drawbacks (limited features, need to purchase an expensive inhaler sensor and need to input data manually at times) leading to a recommendation that a new app be developed to meet the needs of college students (Talavera, D.P., unpublished paper, accessed November 6, 2024).

Betz et al. published a proposed protocol for a scoping review of pediatric asthma apps with the aim of analyzing data on asthma app use both nationally and internationally for individuals aged 0–26 years, with a target audience of low-income and culturally diverse app users.^19^ They planned to include quantitative, qualitative, experimental, and quasi-experimental research. The results of this review have not been disseminated at the time of this publication. Although this review would provide a wealth of evidence about the current state of mobile health apps for young people with asthma, it would not specifically address the concerns of the emerging adult age group.

A scoping review has 4 purposes: a) to broadly describe the scope and characteristics of completed research on a topic, b) to determine whether a more in-depth systematic review should be done, c) to appraise study results and share them with a larger audience, and d) to summarize what is known on the topic.^20^ Given this review's purpose, a scoping review is appropriate. Ultimately, this scoping review will underscore the importance of supporting emerging adults in their progression toward autonomous asthma self-management by identifying the capabilities of current apps as well as recognizing areas that need further research. For adolescents to transition from pediatric to adult care successfully, it is essential to describe the experience and needs of the 17- to 25-year-old population.

## 2 Methods

This review followed the Preferred Reporting Items for Systematic Reviews and Meta-analyses extension for Scoping Reviews (PRISMA-ScR).^21^ Screening started in Rayyan but moved to Covidence after acquisition. Full-text screening and extraction were both completed in Covidence.^22^

### 2.1 Eligibility criteria

Originally, articles selected for this review were to describe the functions of a mobile phone or tablet application (iOS or Android) designed to help emerging adults between 17 and 25 years who are enrolled in college effectively manage asthma symptoms and promote self-care. During full-text screening, the team realized none of the articles specified college enrollment or college students. The team decided to drop the college enrollment criteria in order to have an otherwise relevant set of articles to synthesize. The updated criteria for the full-text screening and extraction were young adults aged 17–25 years utilizing a mobile phone or tablet application (iOS or Android) to manage asthma symptoms. The team adapted the research question to state: *“Are apps available for emerging adults between 17 and 25 years old who are currently enrolled in college to effectively manage asthma symptoms and promote self-care?”* The applications had to be available in the U.S. and in the English language.

Articles were excluded if the applications described (1) were not compatible with iOS or Android operating systems, (2) did not have a primary focus on asthma management in emerging adults, (3) were to share patient information with the provider (versus facilitating asthma self-management by patients), (4) were not available in the U.S. in the English language, (5) were developmentally inappropriate for the audience, or (6) would not open or function indicating technical difficulties in evaluating.

Peer-reviewed journal articles were included if they were published between 2015 and 2023, written in English, involved human participants, and described a mobile application designed to help emerging adults in college manage asthma symptoms. Quantitative, qualitative, and mixed-method studies were included to ensure that as many asthma applications as possible were reviewed. See Appendix A for full description of the research question and inclusion/exclusion criteria.

### 2.2 Information sources and search strategy

Due to the availability of resources and the technology element of this question, PubMed, Scopus and IEEE Xplore were selected and searched. The university’s research and education librarian crafted and executed the strategies for each of the 3 databases. Grey literature was not included in this study. The scoping review team wanted to limit the retrieval to recent literature and the initial search was executed from 2015 through 2022. The searches were updated in March 2023. The strategies utilized keywords and controlled vocabulary (when available) to describe asthma and “mobile app.” Two age group filters, “Young Adult: 19–24 years” and “Adolescent:13–19 years,” were applied to the original 2022 search to locate articles with a relevant population. These filters were omitted in 2023 for a bigger retrieval. The terms “young adult,” “adolescent,” and “teenager” were part of the Scopus search strategy to retrieve articles with the appropriate population. No age group terms were part of the IEEE Xplore search strategy because of the engineering and computer science discipline coverage. However, the “journals” filter was applied to exclude conference proceedings, which did not meet the inclusion criteria.

Copies of each of these strategies can be found in Appendix B.

### 2.3 Search techniques and selection of sources of evidence

The search results were uploaded into Covidence for deduplication. The team completed title/abstract screening, full-text screening, and data extraction in Covidence. Each team member screened the title/abstracts. Two members screened the full text; one member acted as the tiebreaker. Two members completed the data extraction for the final set; the team resolved conflicts as a group.

### 2.4 Data charting process

The data charting process form determined the variables extracted from the selected articles and was developed by the entire team. Each article was then assigned to two reviewers. The reviewers independently used the form to extract the data and discuss their results. Discrepancies in charting were resolved through continued discussion and the data was finalized. The items extracted from each article are listed below.

1. Title of the article
2. Lead author’s contact details
3. Country in which the study was conducted and notes
4. Characteristics of included studies

a. Aim of the study
b. Study design
c. Study funding sources
d. Possible conflicts of interest for study authors
e. Participant age range
f. Gender
g. Enrolled in college
h. Inclusion criteria of study
i. Exclusion criteria of the study
j. Name of the app
k. Purpose of the app (self-care app or intervention app)
l. Method of recruitment of participants
m. Total number of participants recruited for the study
n. Total number of participants that completed the study
o. Description of intervention if present
p. Instruments used to measure change
q. Outcomes reported in the study

### 2.5 Data items

Pairs of scoping team members collectively listed and defined data on article characteristics, explicitly focusing on English-speaking countries where the studies were conducted (United States, Australia and Ireland). The aim of each study was separated out and a description of each was agreed upon. For each article, team members identified and categorized the study design. Depending on the study focus, common designs included qualitative, quantitative, mixed methods, and observational. In addition, the funding source and any possible disclosure of the author's financial interest were noted, if applicable. Participants of each extracted article were described by age range, gender, and whether enrolled in college. A detailed description of the inclusion and exclusion criteria from each article was documented.

As an example, the procedure for recruiting participants was categorized, such as by phone, mail, clinic patients, brochures, online, social media, volunteer, or any combination of the list as well as the number of participants recruited, and how many completed the study. Additionally, if applicable, any intervention was noted and each intervention was described thoroughly. To help maintain transparency and clarity during the research process, the scoping team described if any intervention was used and which instrument measures were used, such as questionnaires, quantitative data analysis using SPSS, or thematic findings from text data. Lastly, the team also listed study outcomes such as changes in asthma symptoms, self-care or self-management practices by the study participants, and knowledge gained.

## 3 Results

### 3.1 Selection of sources of evidence

**Fig. 1:**
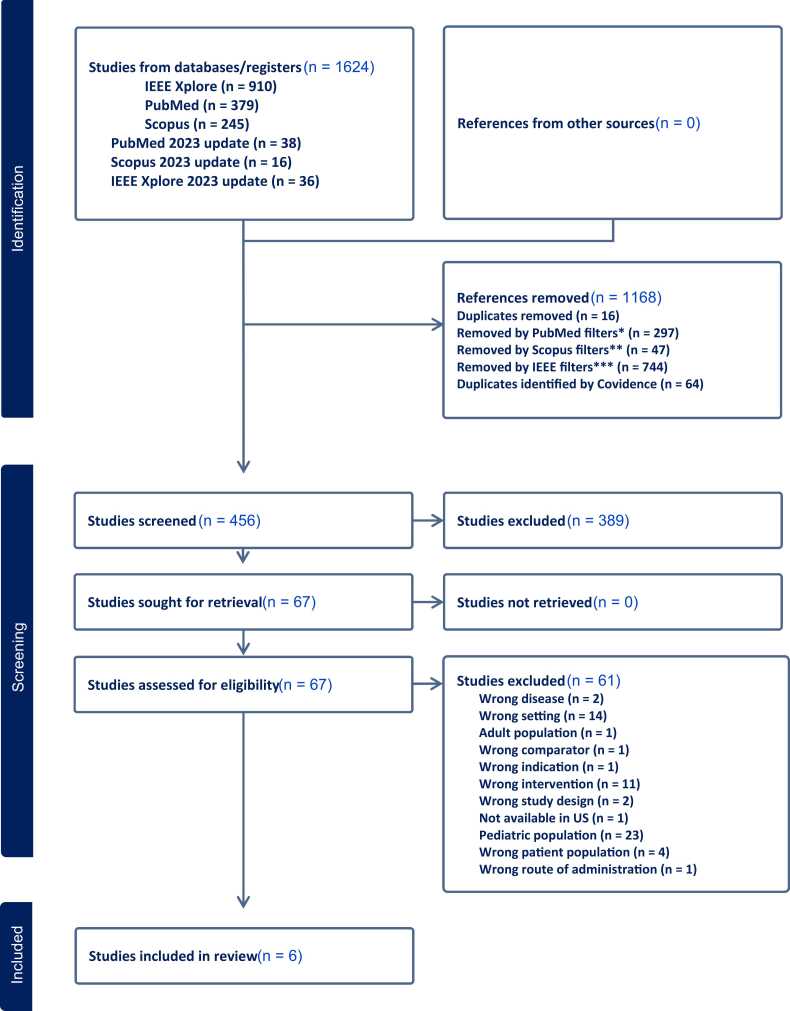
PRISMA-ScR Flow Diagram. *Automatically removed using PubMed filters: 2015 – current (3/1/2023); English Language; Adolescent and Young Adult age selections. ** Automatically removed using Scopus filters: 2015 – March 2023; English Language; Article and Review document type selections. ***Automatically removed using IEEE Xplore filters; 2015 – 2023; Journals. Search update added through 2023.

As seen below in Fig. 1, 1624 articles were retrieved from the original searches in 3 databases (described above) and the 2023 update. Of these, 1168 references were removed before screening after relevant filters (age groups, English language, publication date) were applied. The team screened a total of 456 citations. Of these, 389 studies were excluded after the title/abstract screen, and of the remaining 67 articles, 61 were excluded after full-text screening. The reasons for exclusion are listed below in the flow diagram. Six citations were included in this review. This flow diagram was produced from Covidence.^22^ (Fig. 1).

### 3.2 Selected characteristics of included research studies

Research articles selected for this review (n = 6) were published between 2017 and 2021 in peer reviewed journals. The articles in this review focused on the development and/or testing of three distinct asthma apps and included participants between the ages of 17– 25, although due to sampling techniques, participants of other ages were included as well. Participants were predominantly female with percentages ranging from 42 % to 82 %. Race and ethnicities were reported as White,^23^ Black,^24^, ^25^ White Irish,^23^ Black Irish,^23^ Asian,^23^ Asian Irish,^23^ Hispanic,^24^ non-Hispanic White,^25^, ^26^ Non-Hispanic Black,^24^, ^25^, ^26^ non-Hispanic multiracial,^24^ multiracial,^23^ unknown^24^ and not reported.^25^, ^26^, ^27^ Additional characteristics of the included studies can be found in Table 1.

**Table 1:** Results of individual sources of evidence.

First Author/ Country of Origin	**Year**	**Age Range /Gender**	**Aim**	**Study Design**	**Name of App**	**Description of App**	**Enrolled in College**	**Participants (n) that Completed Study**	**Results**
Peters, et al. (AUS) Funded by Asthma Australia	2017	15 – 24 years; (mean 17.8 years) 12 F 8 M	Explore the psychological experience of young people with asthma and elicit feedback on preferred app features to support users’ psychological needs.	Multi-phased participatory research using mixed methods	Kiss myAsthma one workshop and/or completion of a workbook or both	App with features that support the user’s psychological needs and confidence to self-manage asthma.	6/30 (20 %) enrolled in college	7/20 completed workbook only 13/20 completed workshop and workbook	Young people with asthma tested and gave feedback on features and performance of a prototype app. • Participants recommended adding a mechanism for psychological support for end-users which led to the development of new features. • No p values or effect sizes were reported.
Davis, et al. (AUS) Funded by Asthma Australia	2018	15 – 24 years. (mean age not given) 12 F 8 M	To design a mobile app for young people with asthma and evaluate its effect on confidence and motivation for asthma self-management.	Multi-phased participatory research using mixed methods (pilot study)	Kiss myAsthma 6-week use of app	App allows users to set goals, track symptoms & medication doses, provide emergency instructions, access their AAP, and encourage social connections.	7/20 (28 %) enrolled in college	7/20 completed workbook only 13/20 completed workshop and workbook	• Using participants’ input, an asthma app for young people was developed including tailored notifications, psychosocial support and access to emergency resources. • No p values or effect sizes were reported.
Davis, et al. 2021 (AUS) Funding not clear; Asthma Australia supported school rollout	2021	15 – 24 years (mean age 19.75 years) 5 F 7 M 20 % socially disadvantaged	To evaluate the ability and efficacy of the Kiss myAsthma app to improve self-management in young people with asthma.	Multi- phased participatory research mixed methods (pilot study)	Kiss myAsthma 6-week use of app	Same as Davis (2018)	4/12 (33 %) enrolled in college	9/12 completed baseline and 6 week questionnaire; 5/12 offered goals and strategies; 3/12 contributed to a tool designed to increase intrinsic motivation.	The Kiss myAsthma app demonstrated capacity to support self-management, quality of life and health behavior change in young people with asthma. • At 6 weeks there was a clinically significant improvement in the emotional function domain score of the quality-of-life instrument **(p = 0.043).** • Asthma control was not clinically significant at follow up (p = 0.204) • No effect sizes were reported.
Hsia, et al. (USA) Funded by the American Lung Assn, and AAAAI	2020	7 – 17 years (mean age 10.5 years) 20 M, 19 F 66.7 % Medicaid	To evaluate the effect of ASTHMAXcel Adventures on disease control, asthma knowledge, quality of life, patient satisfaction and health care efficiency.	Single-arm prospective study to evaluate app effectiveness	ASTHMAXcel Adventures (Pediatric version) 6-month use of app	Interactive, gamified app with 11 educational topics and 5 levels of play	Not reported	39/49 completed all 3 evaluation visits	ASTHMAXcel Adventures improved users’ asthma control, knowledge, and quality of life. ED visits and prednisone use were reduced; patients were highly satisfied. • The proportion of controlled asthma increased from visit 1 to visits 2 and 3 (30.8 % vs 53.9 %,( **p = .04)**: 30.8 % vs 59.0 %, (**p = .02)**. • Improved quality of life scores were reported from baseline to visits 2 and 3 (43.3 vs 34.08**, p < .001**; 43.33 vs. 31.74, **p < .001).** • ED visits and prednisone use were significantly decreased from baseline to visits 2 and 3 (ED: 0.46 vs 0.13, **p = .03**; 0.46 vs 0.02, **p = .02**; prednisone use, 0.49 vs 0.13, **p = .02**; 0.49 vs 0.03. **p = .003**). • The authors reported that satisfaction was high with a mean client satisfaction questionnaire score of approximately 30 out of 32 at all visits. • No effect size was reported.
Hsia, et al. (USA) Funded by Genentech & private	2021	18 years & up (mean age 47 years) 49 F 11 M	Describe the development of the ASTHMAXcel app. Compare patient knowledge and satisfaction with the app versus a face-to-face asthma education program.	Prospective pilot study of ASTHMAXcel app	ASTHMAXcel (adult version) One-time use of app	Educational app with personalized algorithms that individualize information for each user.	Not reported	60 (30 app users; 30 controls human educated)	Both the ASTHMAXcel and human- educated groups demonstrated increases in AKQ scores. • Participants in the human-educated group had higher mean AKQ pretest to posttest scores (12.3 vs 14.4) **(p = 0.0002)** than the participants in the ASTHMAXcel group, 9.9 vs 10.5, (p = 0.27.) • The mean AKQ post-intervention for both groups was 2.4 vs 3.9, (**p = .0.007.)** • The ASTHMAXcel group were highly satisfied, scoring on average 27.9 out of 30 maximum points on the satisfaction survey. • No effect size was reported.
Murphy, et al. (IRE) Funded by Irish Research Council	2021	15 – 30 years, (mean age 24.4 years) 101 F 21 M	To engage young adults in a trial on the usability of the AsthmaMD app and determine its efficacy in improving adherence to ICS.	Multi-methods feasibility design	AsthmaMD 2-week use of app	Primary goal: to improve ICS adherence. App features allow education, tracking of medication doses, PFM readings & symptoms and access to AAP.	Not reported	59/122 tertiary education or above	This feasibility study determined that • young adults can be recruited and retained to test the effectiveness of an asthma app in future trials. • AsthmaMD may be effective in supporting ICS therapy adherence in young adults. • No p values or effect sizes were reported.

AAAAI: American Academy of Allergy, Asthma and Immunology; AAP: Asthma Action Plan; AKQ: Asthma Knowledge Questionnaire; ICS: Inhaled Corticosteroid Therapy; PFM: Peak Flow Meter.

### 3.3 Research designs

The research design used and its purpose for each of the six studies chosen will be discussed in this section. Evaluation of the three apps will also be addressed. This section is organized by the title of the app.

#### 3.3.1 Kiss myAsthma^27^

A multi-phased participatory research design was used in this study to provide methods for the direct involvement of users of the app. The purpose of using a participatory design was to compensate for what the authors described as the lack of user-involvement reported in the development of previous health and asthma apps.^27^ Youth with asthma between 15 and 24 years completed a participatory workshop and a workbook about living with asthma, technology use, and the design of an asthma app.

Evaluation: Twenty participants generated 102 artifacts. Theoretical thematic analysis resulted in a set of personal needs, feature ideas, and app characteristics considered relevant by this population for development of an asthma app. For example, psychological factors were considered major influences on quality of life. User evaluation was conducted with nine of the original workshop participants on a high-fidelity prototype app after its initial design.

#### 3.3.2 Kiss myAsthma^25^

This study used a multi-phased participatory research design and mixed methods to pilot test the Kiss myAsthma app with young people with asthma. The purpose of using this design was to develop an evidence-based asthma app tailored to young people’s needs created to optimize user-engagement. The initial app was developed from data collected from 20 asthma patients aged 15–24 years.

Evaluation: Accuracy of the app’s content was determined by three respiratory specialists and two pharmacists. In addition, nine co-designers served as expert reviewers of the prototype app. Participants also scored their satisfaction with the app on a Likert scale questionnaire on items such as content, ease of use, and usefulness for asthma. The authors stated that a clinically sound asthma app was developed for young adults. The authors planned a six-week test of engagement, acceptability, and usefulness of the app in young people not involved in this participatory design.

#### 3.3.3 Kiss myAsthma^26^

A mixed methods approach using quantitative analysis of self-report questionnaires and app usage log data (pages visited, frequency of use, goals set, symptoms recorded) was used. The purpose of using this design was to perform a pilot study to test the engagement, acceptability, and usefulness of the goal-setting smartphone app Kiss my Asthma in young people between the ages of 15–24 years old.

Evaluation: Seventy-five percent of participants completed the baseline and six-week questionnaires. They reported high satisfaction with the app’s content and usability. At six weeks there was a clinically significant improvement in the emotional function domain score of the quality-of-life instrument (p = 0.043). Asthma control was not clinically significant at follow up (p = 0.204).

Participants (42 %) used the app to develop goals and strategies to manage their asthma. The authors concluded that the six-week pilot of the Kiss myAsthma app demonstrated potential to support self-management, quality of life, and health behavior changes in young adults with asthma.

#### 3.3.4 ASTHMAXcel adventures^28^

In the development and evaluation of this app a single-arm study design of patients (7–17 years) with asthma was used. The purpose of using this design was to assess the impact of ASTHMAXcel Adventures, a gamified guidelines-based, pediatric version on asthma control, knowledge, health care utilization, and patient satisfaction. There are eleven chapters with five levels of play designed to reflect educational content from British and Scottish Asthma Guidelines. Educational content was reviewed by a team of asthma physicians, educators, and a behavioral scientist to ensure consistency with these guidelines.

A group received asthma education from the ASTHMAXcel Adventures mobile app from an iPad tablet at an Asthma Center at baseline during a 10-week period. Participants completed questionnaires to assess asthma control, asthma knowledge, and patient satisfaction at 4 months (visit 2) and 6 months (visit 3).

Evaluation: Thirty-nine participants completed the study. Asthma control, asthma knowledge, and quality of life significantly improved while ED visits and prednisone use significantly decreased from baseline to post visits.^24^ Specifically, the proportion of controlled asthma increased from visit 1 to visits 2 and 3 (30.8 % vs 53.9 %, p = .04; 30.8 % vs 59.0 %, p = .02) largely seen in boys. Mean asthma knowledge score measured as part of asthma illness representation scale increased from baseline to post-intervention, with sustained improvements at visit 2 and visit 3 (3.55 vs 3.76, p < .001; 3.55 vs 3.80, p = .001; 3.55 vs 3.99, p < .001). Improved quality of life scores were reported from baseline to visits 2 and 3 (43.3 vs 34.08, p < .001; 43.33 vs. 31.74, p < .001). ED visits and prednisone use were significantly decreased from baseline to visits 2 and 3 (ED: 0.46 vs 0.13, p = .03; 0.46 vs 0.02, p = .02; prednisone use, 0.49 vs 0.13, p = .02; 0.49 vs 0.03. p = .003). The authors reported that satisfaction was high with a mean client satisfaction questionnaire score of approximately 30 out of 32 at all visits.

#### 3.3.5 ASTHMAXcel^26^

In the development of this app version for adults, a prospective pilot study design was used to test ASTHMAXcel among (n = 30) adult patients (18 years and older) at an asthma clinic compared to those (n = 30) receiving asthma education face-to-face from a human educator. All participants were evaluated in relation to asthma knowledge, process outcomes such as time spent completing the program, and patient satisfaction.

Evaluation: The participants in the human educator group had a higher mean asthma knowledge questionnaire score (AKQ) pretest to posttest (12.3 vs 14.4, p = 0.0002) than the participants in the ASTHMAXcel group (9.9 vs 10.5, p = 0.27). However, the mean AKQ post-intervention score for both groups was significantly higher (2.4 vs 3.9, p = .0.007). Patients were highly satisfied in the ASTHMAXcel group, scoring on average 27.9 out of 30 maximum points on the satisfaction survey.

#### 3.3.6 AsthmaMD^23^

A multi-methods feasibility design was used to determine the probability of recruiting and retaining young adults (18–30 years) with asthma to participate in a trial and the usability, acceptability, and feasibility of using the Asthma MD app was assessed to support adherence to inhaled corticosteroids (ICS) for 2 weeks in this population. Primary outcomes included evaluation of participant recruitment and retention, and the usability, acceptability, and feasibility of using AsthmaMD.

Evaluation: Of the 122 young adults recruited, 59 (48.4 %) completed the study. The AsthmaMD app received a score of 63.1/100 (SD 20.1) on the System Usability Scale. Two main themes were identified regarding the user’s experience with the app: (1) learning how to use the app to meet individual needs and (2) benefits and relevance of using the app on adherence to ICS therapy. Authors recommended providing more accessible information on the use of the app and simplifying the medical language used in the app. Additional information on the selected studies is found in Table 1.

### 3.4 Themes generated from the extracted data

In analyzing the data during this review, several themes were discovered. The themes provide a better understanding of the evidence found, highlight some of the significant findings, and underscore their relevance to the research question: *“Are apps available for emerging adults between 17 and 25 years old who are currently enrolled in college to effectively manage asthma symptoms and promote self-care?”*

#### 3.4.1 Theme #1 population, education and age range

The first theme focuses on the population, emphasizing participants' educational background and age range.

##### 3.4.1.1 Population and age

Each extracted article focused on a specific age range, with the majority emphasizing younger populations, specifically children and adolescents. The age span for all publications reviewed was 7 years to adult. Although several targeted articles included participants within the 17–25 age range specified in the inclusion criteria, most studies also included younger children.^24^, ^25^, ^26^, ^27^ Other authors focused on adult participants.^23^, ^28^

##### 3.4.1.2 Education

Peters et al.^27^ reported that 6 of 30 participants were enrolled in college, while Davis et al.^26^ indicated that 4 of 12 participants in one study and 7 of 20 in a second study were college students.^25^ The participants were young people with diverse educational backgrounds. Three articles specifically mention the percentage of college student participants which ranged from 20 % – 33 %. Although Murphy et al.^23^ used methods to recruit university students, they did not report the number of participants currently attending college. Additional information about the proportion of college student participants is found in Table 1.

#### 3.4.2 Theme #2 app design and purpose

The next theme highlights the design elements and purpose of the selected articles included in the scoping review.

##### 3.4.2.1 Pilot study design

Several articles described pilot studies of newly developed apps. The ASTHMAXcel Adventures app, which aims to improve pediatric participants’ asthma control via use of gamified educational material and self-logging of asthma symptoms, was pilot tested on 39 participants.^28^ The adult iteration of the app, ASTHMAXcel, which has an educational focus, was pilot tested on 60 adults to compare its effectiveness with asthma education received from asthma educators.^24^ A pilot study on the AsthmaMD app was designed to improve treatment compliance with inhaled corticosteroid medications utilizing regular medication reminders sent to users.^23^ This app included educational content, tracking logs, and a provider communication feature. These apps were designed to be easy to use, increase asthma self-management knowledge, and engage users to improve asthma outcomes.

##### 3.4.2.2 Gamification design

Gamification includes game-like design elements to enhance an app user's experience and motivate their desire to learn. It typically includes a point system with a leaderboard, a way to earn badges, and offers challenges to encourage participation and improve outcomes.^29^ App developers strived for increased adherence through a mobile gamification app called ASTHMAXcel Adventures, which the authors reported improved outcomes such as reduced ED visits and prednisone use.^28^ This app includes several gamification elements reported to support asthma self-management in the 7–17-year-old population.^28^ The gamification elements were modified to be age-appropriate to engage the broad age range (7–12 and 13–17 years). For example, the tasks were simplified for the younger audience and made more complex for the older participants. In addition, the avatars were adapted to the developmental level and the common interests of each age group. Some of the gamification elements include learning modules, videos, quizzes, individual feedback, and health recommendations. The gamification features aim to improve the participants' knowledge surrounding asthma and manage symptoms to reduce the need for emergency interventions.

##### 3.4.2.3 Participatory design

Other publications reviewed stressed the importance of engaging the end-user through participatory research design.^25^, ^26^, ^27^ Participatory research design involves collaborating with participants during the research process to tackle similar questions or concerns.^30^ Through multi-phased mixed methods studies, researchers examined the Kiss myAsthma app, where young people were asked to participate in concept development, giving feedback and then brainstorming specific app features.^25^, ^26^, ^27^ Those who participated were asked whether the app was usable and relevant in “think aloud” sessions and if it fostered engagement.^25^ Focus groups were utilized for brainstorming sessions to define the preferred features for an asthma self-management prototype.^27^ Interestingly, participants emphasized the importance of addressing the psychological needs of young people aged 15–24 years and the challenges this population faces daily living with asthma without being prompted.^27^

##### 3.4.2.4 Feasibility design

Similar to participatory design, a feasibility design asks for the participants' opinions to better understand whether an intervention is practical and whether they are willing to participate in a small-scale pilot study before a project moves forward.^31^ Although participatory and feasibility designs vary, as they have different outcomes, both require the collaboration of participants to obtain results.^32^ For example, Murphy et al.^23^ explicitly stated that they wanted to uncover whether it would be feasible to study the efficacy and effectiveness of the AsthmaMD app in a future study.

#### 3.4.3 Theme #3 user-friendliness of apps

The third theme uncovered by the scoping review team highlighted the user-friendliness of the various apps. Participants in these articles commented on the ease of navigating the app and its overall usability in terms of behavior changes and other quality-of-life measures. In particular, user-friendliness of the apps was evaluated by exploring their impact on asthma self-management, health behavior changes, and other quality of life measures.^23^, ^24^, ^25^, ^26^, ^28^ Methods to enhance user-friendliness included collecting feedback from young people to design an app that intentionally appealed to a specific age group,^25^, ^26^ incorporating gamification to increase user adherence,^28^ and focusing on strategies to improve medication adherence.^23^

#### 3.4.4 Theme #4 focus on self-management or education

The next theme unearthed by the scoping team emphasizes the educational tools included within the apps to enhance the user’s experience and improve health outcomes.

#### 3.4.5 Educational tools

The importance of using educational tools within an app to enhance asthma knowledge and support improved health outcomes was evident in the ASTHMAXcel apps.^24^, ^28^ Asthma education was provided in ASTHMAXcel Adventures, a gamified app geared toward the pediatric asthma population. ASTHMAXcel Adventures also integrated educational videos to improve understanding of the pathophysiology of the illness and how medication adherence and proper technique improve symptoms. Lastly, the participants' knowledge and quality of life were assessed with a point system that rewards users as they learn. The ASTHMAXcel app used personalized algorithms and videos to educate adult participants.

#### 3.4.6 Self-management tools

All reviewed apps were designed to improve asthma self-management skills and promote medication adherence and symptom recognition. For instance, Davis et al.^25^ used a participatory design for the Kiss myAsthma app, which included tracking tools and social media components that connect users with other asthmatics for emotional support. In another study, Davis et al.^26^ explored how to tailor these features for young people. In addition to asthma education, symptom logging for adults was included in the ASTHMAXcel app,^24^ and integrated gamification was used in the ASTHMAXcel Adventures app to help children set asthma goals. Through interactive games and quizzes, children were taught about asthma triggers and self-management strategies.^28^ Murphy et al.^23^ designed an app emphasizing inhaled corticosteroid adherence and prevention strategies. Finally, Peters et al.^27^ participatory design uncovered the importance of young people's self-management preferences, such as the need for reminders and stress management tools and the importance of addressing psychological factors in app design.

Building on these insights, the AsthmaMD app that Murphy et al.^23^ examined was based on Abraham and Michie’s theory of behavior change technique taxonomy (BCTTv1).^33^ This app used 10 of the 26 behavior change techniques thought to promote self-management behaviors in the design of the app. Similarly, the taxonomy of behavior change theory has been used to develop apps to manage other chronic illnesses in emerging adults. Godino et al.^34^ evaluated social and mobile app tools for weight loss in 404 overweight and obese young adults in the college setting in relation to BCT. Another author evaluated commercially available medication adherence apps to determine if app frameworks aligned with the principles of behavior change technique to promote medication compliance in 20 adolescents and young adults with chronic illnesses.^35^

#### 3.4.7 Theme #5 limited long-term studies

The final theme brought forth by the scoping review team underscores the significant gap in the literature, specifically, the lack of long-term studies evaluating the sustainability and long-term impact of these apps. Only the pilot study by Hsia et al.^24^ collected data for an extended time frame (6 months) which provided a needed long-term prospective on the frequency of app usage as a form of ongoing support for asthma patients. The remaining five articles were short-term (single interactions up to 6 weeks) which makes sustainability of these apps unknown. Decreased use of apps over time was noted in even the short-term trials, so longer-term studies are needed to determine whether asthma apps are a successful, sustainable intervention to support ongoing asthma self-management. Table 2 provides detailed information on each theme.

**Table 2:** Summary of evidence extracted themes.

Major Theme	Subtheme	Peters et al., 2017	Davis et al., 2018	Davis et al., 2021	Hsia et al., 2020	Hsia et al., 2021	Murphy et al., 2021
1) **Population, Education, and Age Range**							
	Population and age	x	x	x	x	x	x
	Education			x			x

2) **App Design and Purpose**							
	Pilot study design				x	x	x
	Gamification				x	x	
	Participatory design	x	x	x		x	
	Feasibility design						x

3) **User-friendliness of App**			x	x	x	x	x

4) **Focus on Self-Management & Education**							
	Educational Tools				x	x	
	Self-Management Tools	x	x	x	x	x	x

5) **Gap in Literature: Limited long-term studies**		x	x	x	x		x

## 4 Discussion

In this scoping review, 456 research studies exploring asthma apps published between 2015 and 2023 were analyzed for their applicability to college students between 17 and 25 years with asthma. The research team sought to answer the question: *Are apps available for young adults between 17 and 25 years old who are currently enrolled in college to effectively manage asthma symptoms and promote self-care?* The results of this scoping review revealed that studies examining the experience of this emerging adult population and the distinct obstacles that they face when the transition to asthma self-management occurs while in college and away from their usual support systems are lacking. The review identified six articles involving participants within age ranges overlapping the target 17–25-year college-aged group. Participants in the Peters et al. and Davis et al. articles were 15–24 years old^25^, ^26^, ^27^ while the population in Murphy et al. spanned 15–30 years.^23^ Hsia et al. included 7- to 17-year-olds in their 2020 study and participants 18 years and older (M= 47 years) in their 2021 study^24^, ^28^ This age variability, coupled with the lack of studies specifically targeting the challenges of asthma self-management among college students, highlights a significant gap in the literature.

The authors of the three apps were mindful to use features to gain and retain the attention of young adults. For example, user-friendliness was frequently emphasized, with app features such as tailored feedback and gamification (e.g., ASTHMAXcel Adventures), improved self-management and quality of life, though not specifically for college-age users.^23^, ^24^, ^25^, ^26^, ^27^, ^28^ One app prioritized educational tools (e.g., videos in ASTHMAXcel) and self-management features (e.g., symptom tracking in Kiss myAsthma, and medication reminders in AsthmaMD), fostering knowledge and adherence, but again lacking a college-specific focus.^23^, ^24^, ^25^, ^26^, ^27^, ^28^

College students with asthma face barriers including increased independence, academic pressures, variable access to health care, and living with the unpredictable nature of asthma away from their support systems.^36^ Additionally, asthma control challenges for the emerging adult population include medication adherence, understanding the severity of their condition, feelings of embarrassment associated with using asthma medication, managing life demands, the cost of medications, and difficulty obtaining medication refills.^37^ Previous studies also report that both emerging adults and their parents are mostly dissatisfied with the process of transitioning from pediatric to adult health care,^3^ suggesting that more support is needed for this vulnerable population during the transition process.

The research uncovered in this scoping review captures the age range of most college students but not the developmental and transitional needs specific to their role as college students.^23^, ^24^, ^25^, ^26^, ^27^, ^28^ Mobile apps for asthma management that contain features such as medication reminders (AsthmaMD), symptom tracking, and access to Asthma Action Plans (Kiss myAsthma), as well as disease-specific education (ASTHMAXcel), have the potential to improve asthma outcomes but must be tailored to the end-user’s needs.

Asthma creates a range of financial and health problems, which make it especially difficult for college students to handle their health needs and academic responsibilities independently.^25^, ^27^ The Theory of Being Unprepared^12^ suggests that emerging adults also often depend on others, primarily their mothers, to manage their asthma throughout their high school years. Consequently, some students maintain this dependence as they transition to college, leading to insufficient asthma self-management skills and a lack of preparedness.^12^ Asthma apps contain valuable information and features, which may make them a viable tool to bridge the gap between parent-managed and self-managed asthma.

These findings underscore the potential applicability of asthma apps for a successful transition to self-managed asthma but reveal a critical gap in tailored interventions addressing the unique developmental, psychological, academic, and health care challenges faced by college students.

### 4.1 Research gaps and future directions

The literature lacks systematic investigations that track app effectiveness over an extended period for this population. The Davis et al. pilot study (Kiss myAsthma) reported significant improvements in asthma quality of life;^26^ however, Hsia et al. (ASTHMAXcel) conducted the only study that tracked participants for six months to assess impact of extended app use.^28^ Future research should include randomized controlled trials on a larger scale to demonstrate long-term efficacy of this technology. App developers could also adopt participatory design methods (as done with Kiss myAsthma) by working with college students to design futuristic apps that incorporate wearable device features and contain qualities that they may find useful, such as academic calendar compatibility.

Future research should focus on developing college-specific asthma app features that address barriers stemming from variable schedules, psychological and social pressures, and restricted health care provider availability that the college student population faces. It is important to engage this population in the development of an app to identify the specific barriers that they encounter when transitioning to college and evaluate whether phone self-management applications could, in part, serve as support for emerging adults with asthma during this transition.

## 5 Strengths and limitations

This scoping review, encompassing investigation of 456 studies, provides information to map the existing asthma app landscape due to its comprehensive analysis. The thematic insights gained from this review serve as a call for additional research focused on the emerging adult age group attending college not previously examined. Much can be learned from other apps on the market that assist the emerging adult population with self-management of other chronic illnesses, such as diabetes, mental health and obesity.^34^, ^38^, ^39^

Limitations included small sample sizes, self-report bias, and unequal access to mobile phones for some disadvantaged groups, making generalizability more difficult. As asthma management remains a significant economic burden on health care, mobile asthma app development is essential to enhance care for the emerging adult population and align with their increased desire for electronic resources, including health media.

Given the economic impact of asthma on health care costs, mobile apps may be a valuable tool for emerging adults that align with the growing use of digital health tools to enhance care and support the transition to independent self-management. These findings are restricted to English-language publications from 2015 to 2023 and exclusion of studies beyond this period. This review presented multiple study designs alongside diverse participant demographics, which made it challenging to conduct straightforward comparisons.

## 6 Conclusion

The available research lacks essential information about asthma mobile apps that cater to college students. The identified themes of psychosocial support, self-management, and education provide important findings yet show that college students require special interventions to address their unique challenges. This scoping review emphasizes the need for increased innovation in app design and rigorous follow-up research to develop and evaluate apps that specifically target college students, empowering this population to manage their chronic condition during their transition to college and thus foster greater independence.

## Ethics statement

The work that we are submitting has not been published previously and the article is not under consideration for publication elsewhere. All authors approve of the publication of this article. If accepted, the article will not be published elsewhere in the same form, in English or any other language, including electronically, without the written consent of the copyright holder.

## CRediT authorship contribution statement

**Elizabeth Huggins:** Writing – review & editing, Writing – original draft, Methodology. **Susan McNamee:** Writing – review & editing, Writing – original draft. **Margaret C. Delaney:** Writing – review & editing, Writing – original draft. **V. Ann Andreoni:** Writing – review & editing, Writing – original draft. **Barbara Velsor-Friedrich:** Writing – review & editing, Writing – original draft, Supervision.

## Funding

This research was conducted without financial support or external funding.

## Declaration of Competing Interest

The authors declare that they have no known competing financial interests or personal relationships that could have appeared to influence the work reported in this paper.

## Figures and Tables

**Table:** 

**Inclusion Criteria Applied for Search**	**Comments**

App must be available for download on iOS or Android platform	iOS or Android
Able to download onto a smartphone or tablet	Phone or tablet
Written in English (app discussed)	English
Available in the United States (app discussed)	U.S.
App primary focus on asthma with respect to either education, support, or self-management	Asthma focus
College enrollment	Yes – enrolled in college
Target age	17–25 yrs
**Exclusion Criteria Applied for Search**	**Comments**
Not available through an approved device (iPhone or Android)	Not iPhone or Android
Disclaimed for non-health related purposes	Non-health related
App not available in U.S. (United States)	Not U.S.
Could not be used because of technical problems	Technical problems
Primary purpose of app is to interface with the provider	Only provider interface
Irrelevant	Irrelevant
Developmentally inappropriate	Inappropriate
Wrong disease process	Wrong disease

## Data Availability

The authors reviewed data collected by other authors and do not have permission to share their data. We can share information about how the review process was conducted

## Appendix 1

Asthma Scoping Review of Literature

Inclusion/Exclusion Criteria

Research Question: *Are apps available for young adults between 17 and 25 years old who are currently enrolled in college to effectively manage asthma symptoms and promote self-care?*

(adapted from Huckvale et.al, 2012, 2015)
